# Patterns of Romantic Relationship Experiences and Psychosocial Adjustment From Adolescence to Young Adulthood

**DOI:** 10.1007/s10964-020-01350-7

**Published:** 2020-11-16

**Authors:** Tita Gonzalez Avilés, Christine Finn, Franz J. Neyer

**Affiliations:** Institut für Psychologie, Friedrich-Schiller-Universität, Jena, Germany

**Keywords:** Romantic relationships, Adolescence, Young adulthood, Psychosocial adjustment, Longitudinal

## Abstract

Engaging in a romantic relationship is a key developmental task of adolescence and adolescents differ greatly in both the age at which they start dating and in how romantically active they are. These differences in romantic relationship experiences could be relevant for adolescents’ short- and long-term psychosocial adjustment. The present study describes the diversity of relationship experiences during adolescence and examines their connection to psychosocial adjustment in adolescence and young adulthood. *N* = 2457 adolescents (49.3% female) from a German representative longitudinal study provided information on their relationship experiences between the ages 10 and 20, as well as on their psychosocial adjustment. Data were collected via annual assessments starting in 2008 at age *M* = 16.50 years (*SD* = 0.88) through young adulthood (*M* = 25.46, *SD* = 0.87). Latent profile analysis identified three romantic involvement groups: late starters, moderate daters, and frequent changers, which were further compared to adolescents without any romantic experiences (continuous singles). Growth curve analyses indicated that continuous singles reported lower life satisfaction and higher loneliness than the moderate daters in adolescence and young adulthood. The continuous singles were also less satisfied with their life in young adulthood and felt more lonely in both adolescence and young adulthood compared to the late starters. The findings of the study suggest great variability in adolescents’ romantic relationship experiences and point toward the developmental significance of these experiences for short- and long-term well-being.

## Introduction

Romantic relationships are a major aspect of adolescents’ lives, as adolescence is often when romantic experiences and relationships first take place (Seiffge-Krenke 2003). These new forms of social relationships provide an important context to develop skills needed for future relationships, and represent a source of support, companionship, and intimacy (Collins et al. 2009). Although often described as exploratory and unstable (Collins et al. 2009), several recent studies have recognized variability in adolescent romantic relationships (Boisvert and Poulin 2016; Connolly et al. 2013; Orpinas et al. 2013): While some adolescents indeed have many relationships of short duration, others have one stable relationship over time, others do not date at all, or start dating at a later age. Due to the developmental significance of romantic relationships during adolescence (Collins et al. 2009), these differences in relationship experiences may be associated with both short- and long-term adjustment. The purpose of the present study is to gain a greater understanding of the variety of romantic relationship experiences during adolescence, and to study the relevance of these experiences for psychosocial adjustment from middle adolescence to young adulthood.

### Romantic Relationships in Adolescence

First romantic relationships are typically established during middle adolescence, around the age of 14–15 years (Collins et al. 2009). At age 15, 43% of a German sample have reported currently being in a relationship (Seiffge-Krenke 2003). While first relationships are often of short duration, they become more stable over the course of adolescence. Seiffge-Krenke (2003), for example, found mean relationship duration to increase between ages 15 and 21 from 5.1 to 21.3 months. Over the course of adolescence, most people report having had around three relationships (Boisvert and Poulin 2016). Despite these general trends, there is substantial variability in when adolescents initiate romantic relationships and how romantically active they are.

Differences in romantic experiences can be described by relationship patterns, which take into account multiple features of romantic involvement and consider them in combination. Multiple studies have used a person-centered approach to describe relationship patterns during adolescence (Boisvert and Poulin 2016; Connolly et al. 2013; Orpinas et al. 2013). These studies typically find that adolescents who have dated at least once can be categorized into three to five groups based on whether they had early or late entry into their first relationship, few or many romantic partners, and little or much time spent in relationships.

Most previous work in this area, however, is limited in several aspects. Previous studies have often neglected the existence of the substantial proportion of adolescents who do not date at all. Research on adult populations suggests that around 20% of young adults have never been involved in a romantic relationship before the age of 25 (Wagner et al. 2015). Excluding those who do not date results in an incomplete picture of the variety of romantic involvement. Second, many studies have either described their groups based on participants’ current relationship status (Connolly et al. 2013; Orpinas et al. 2013) or on both number of partners and total years spent in relationships (Boisvert and Poulin 2016). In contrast, examining more aspects of romantic involvement simultaneously would lead to a more accurate picture. In addition, number of years spent in romantic relationships might be too imprecise to assess relationship duration, especially in an age span generally characterized by short relationship duration. Months instead of years spent in relationships might be better suited to capture relationship duration in adolescence. Third, many of these studies have focused on either the first (ages 12–17; Connolly et al. 2013) or second half of adolescence (ages 16–24; Boisvert and Poulin 2016), and studies covering variations in romantic involvement during the whole period of adolescence from early through late adolescence are still missing. Finally, most studies have relied on small and non-representative samples (for an exception, see Connolly et al. 2013), which may limit the generalizability of their findings.

### Romantic Relationships and Psychosocial Adjustment in Adolescence

Engaging in romantic relationships has long been seen as an important developmental task of adolescence. Furman and Shaffer (2003), for example, theorized that a romantic partner can serve as attachment figure that the adolescent can turn to for friendship, support, intimacy, and sexuality. In addition, being romantically involved can be beneficial for key developmental tasks of adolescence, including identity and sexual development, becoming more independent from one’s parents, and forming close relationships with peers. Indeed, some studies point towards the benefits of engaging in dating in adolescence, as those who engage in romantic relationships report higher self-esteem in middle and late adolescence (Ciairano et al. 2006) and are generally perceived as more popular by their peers (Miller et al. 2009).

However, other theoretical approaches have suggested that dating during adolescence can have negative consequences for the well-being of at least some adolescents, proposing either early age or non-normativity as the main reason. In his theory of psychosocial development, Erikson (1968), proposed that forming close and intimate romantic relationships is a developmental task that is more relevant in young adulthood, while identity development, instead, is the primary task in adolescence. From this perspective, a preoccupation with dating before having established a personal identity could be problematic for future adaptation and function. Romantic relationships in adolescence may also be emotionally challenging and overwhelming as they require levels of attention, communication, and problem-solving skills that may not yet be fully developed (Davila 2008). Another theoretical approach suggests that getting involved either much earlier or much later than one’s peers can be problematic for later adjustment (Connolly et al. 2013), while adolescents who conform to norms (i.e., who get romantically involved in a developmentally typical time) are more likely to be better adjusted. This is because those who engage in behaviors earlier or later than the norm might receive more negative social sanctions and fewer social resources, which could lead to persistent developmental disadvantages (Elder et al. 2003).

Indeed, studies have shown that those who start dating in early adolescence show more depressive symptoms (Natsuaki and Biehl 2009), and more aggressive and delinquent behaviors (Connolly et al. 2013) than those starting later in adolescence. Entering into one’s first relationship later than one’s peers, however, was also found to be associated with more social anxiety (La Greca and Harrison 2005) and lower social competences (Davies and Windle 2000). In addition, those who do not date at all during their adolescence experience greater social dissatisfaction (Beckmeyer and Malacane 2018) and lower self-esteem (Ciairano et al. 2006) than those who report having had at least one relationship. In general, more studies have investigated the effect of getting romantically involved at an early opposed to a later age.

Together, these frameworks and previous findings suggest that dating can be beneficial for adolescents’ well-being, when initiated at a normative age and to a normative extent. While most studies have focused on the age of one’s first romantic relationship as a main contributor to adolescent adjustment, less is known about the potential role of number of relationships and total time spent in romantic relationships as it pertains to well-being. Being romantically over-involved, very sporadically involved, or not at all involved could present additional risks to psychosocial adjustment. In particular, the combination of these aspects of romantic relationships (i.e., age, number, and duration) could be relevant. Davies and Windle (2000), for example, found that early age of first relationship was associated with fewer problematic behaviors when participants had fewer as opposed to more partners.

### Psychosocial Adjustment From Adolescence Through Young Adulthood

Previous studies on the development of psychosocial adjustment from adolescence through young adulthood have yielded inconsistent results. Some point towards increases in self-esteem (Orth et al. 2018) starting in late adolescence, but others suggest decreasing life satisfaction (Goldbeck et al. 2007) and increasing depressive symptoms (Thapar et al. 2012) from middle adolescence through young adulthood. Lastly, some studies find no change in life satisfaction (Baird et al. 2010) or loneliness (Mund et al. 2020) during this period. However, large differences in the amount and direction of change suggest a variety of trajectories that may be partly explained by the diverse relationship experiences had during adolescence.

Davies and Windle (2000), for example, found that an increase in adolescents’ number of romantic partners was associated with increased emotional distress over a period of one year. Conversely, abstaining from dating was associated with decreased self-esteem over a period of eight years in a sample of young adults (Lehnart et al. 2010). However, few studies have investigated the long-term impact of adolescents’ dating experiences on young adults’ adjustment. Those studies that do exist have found that adolescents who reported a higher number of relationship partners and more frequent dating were more likely to show poorer emotional well-being (Longmore et al. 2016; Szwedo et al. 2015) in young adulthood. However, exactly how the development of psychosocial adjustment is affected by multiple aspects of adolescent romantic involvement is still unclear.

## Current Study

The current study utilizes data from a large and nationally representative study following adolescents from the age 16 until 25 to address three major questions. First, the study seeks to obtain a rich picture of the diversity of relationship experiences in adolescence. Thus, within-individual combinations of the following three aspects are examined: age of first relationship, number of romantic partners and number of months spent in relationships between the ages of 10 and 20. Based on previous findings, it was hypothesized that those who have had at least one romantic relationship during their adolescence could be meaningfully divided into three to five groups. This study also hypothesized an additional group of adolescents who have no dating experiences. Second, the study examines whether the previously identified groups differed in their psychosocial adjustment during adolescence, and examines both problematic (loneliness and depressive symptoms) and beneficial (life satisfaction and self-esteem) dimensions of adjustment. While these different constructs all reflect psychosocial adjustment, each captures a unique aspect: *Life satisfaction* reflects a cognitive appraisal of one’s life, *self-esteem* the subjective evaluation of one’s self (a core aspect of one’s self-concept), and *depressive symptoms* a stable tendency for experiencing negative emotions (Diener et al. 1999). *Loneliness* represents a subjective perception of one’s social relationships as deficient in terms of quality or quantity (de Jong Gierveld 1998). Based on previous findings and theoretical considerations discussed in the introduction, it was hypothesized that groups characterized by either high cumulative romantic experiences in adolescence (i.e., early age, high number of partners, large amount of time spent in relationships) or little to no romantic experiences during that time would both show lower psychosocial adjustment than groups with more normative experiences. Finally, the study explores how these groups develop in their psychosocial adjustment through young adulthood. With limited research to guide predictions, no specific hypothesis about the impact of romantic experiences on development through young adulthood was made. However, based on the few studies that found lasting effects of romantic involvement during adolescence on later mental health, differences that were found in adolescence were anticipated to persist through young adulthood.

## Methods

### Data and Procedure

Data were drawn from the German Family Panel *pairfam* (Release 10.0; Brüderl et al. 2019), a nationally representative longitudinal study from Germany. Started in 2008, the panel study currently comprises 10 waves of data on an initial sample of *N* = 12402 participants from three birth cohorts born in the years 1971–1973, 1981–1983, and 1991–1993. The representative sample was drawn from register data, and participants are interviewed annually using computer-assisted personal interviews (CAPI) as well as self-administered computer-based assessments (CASI). Participants are provided a cash incentive of 10 Euro for participating in each interview (see Huinink et al. 2011, for full data description).

### Participants

For the purpose of the current study, only data from the youngest birth cohort (1991–1993; *N* = 4338) were used. *N* = 2534 (58.4%) of them participated in the study until they were at least 20 years old and were thus able to provide information regarding their relationship histories between ages 10 and 20. *n* = 77 participants were excluded from analyses because their relationships were either all very short (i.e., <1 month), they exclusively had cyclical relationships, or they provided insufficient information on relationship initiation or end.

The final sample comprised *N* = 2457 participants (49.3% women) with a mean age of 16.50 years (*SD* = 0.88) at the first wave and *M* = 25.46 years (*SD* = 0.87) by the tenth wave. Additional information on sample size and age for all waves can be found in Table 1. The majority of the participants (79.4%) were German natives with no migration background, 5.4% were half-German, 5.2% were ethnic German immigrants, 3.4% had a Turkish migration background, and 6.6% stated that they had another non-German background. Most participants were attending high school during the first wave (44.2%), followed by secondary school (20.3%) and vocational training (10.5%). The remaining 25% were enrolled elsewhere (e.g., secondary general school). *N* = 1915 (77.9%) reported having had at least one romantic relationship between the ages of 10 and 20, while *n* = 542 (22.1%) were not romantically involved during this time. 98.8% of the adolescents identified as heterosexual during the first wave.

**Table 1:** Description of study sample and overview of psychosocial adjustment assessment

Variable	W1	W2	W3	W4	W5	W6	W7	W8	W9	W10
Sample size	2457	2375	2379	2323	2228	2077	1813	1669	1543	1397
% female	0.49	0.49	0.49	0.49	0.49	0.49	0.50	0.50	0.50	0.51
Age										
*M*	16.50	17.54	18.55	19.52	20.48	21.44	22.44	23.45	24.44	25.46
*SD*	0.88	0.88	0.89	0.88	0.88	0.88	0.87	0.87	0.87	0.87
Assessment^a^										
Life satisfaction	x	x	x	x	x	x	x	x	x	x
Self-esteem		x	x	x	x	x	x	x	x	x
Loneliness				x	x		x	x	x	x
Depressive symptoms		x	x	x	x	x	x	x	x	x

W1–W10 = measurement occasions 1–10

^a^Whether variable has been measured in particular wave

To explore attrition effects, individuals who did not participate in the last wave were compared to those who did on psychosocial adjustment at the first assessment. No significant differences were found in neither of the aspects, indicating no systematic attrition and that data were missing at random.

### Measures

#### Romantic involvement

Romantic involvement was assessed by means of an event-history calendar (EHC; Belli and Callegaro 2009), a widely used instrument based on a graphical time frame that uses visual cues to facilitate autobiographic memory retrieval. The EHC was implemented for the first time in Wave two, where participants reported on current and past romantic relationships. This information was updated in each subsequent wave, providing a full description of their entire romantic relationship history. Based on this information, three variables were created: Age of first relationship, number of relationships, and total months spent in relationships between the ages of 10 and 20.

##### Age of first relationship

A variable reflecting the age of first relationship was created based on the age at which participants first reported having had a romantic partner. This variable was only calculated for those who reported having had at least one relationship by age 20.

##### Number of relationships

The number of different romantic partners was calculated by adding up the number of partners listed between the ages of 10 and 20.

##### Months spent in relationships

This variable was compiled by adding up the number of months spent in romantic relationships between the ages of 10 and 20, independent of the number of partners.

#### Psychosocial adjustment

Different aspects of psychosocial adjustment were assessed over multiple waves. Assessments differed in number of items, time of first assessment, mode, and measurement interval (see Table 1). Life satisfaction was assessed by means of the CAPI, and self-esteem, loneliness, and depressive symptoms with the CASI.

##### Life satisfaction

Life satisfaction was assessed with one item adapted from the German Socio-Economic Panel (i.e., “All in all, how satisfied are you with your life at the moment?”) ranging from 0 (*very dissatisfied*) to 10 (*very satisfied*). This instrument was found to be reliable with a retest reliability of around 0.70 (Lucas and Donnellan 2012) and to perform very similarly to its multiple-item counterpart (Cheung and Lucas 2014). The correlations between life satisfaction and the other psychosocial adjustment variables at Wave four (self-esteem: *r* = 0.43, loneliness: *r* = −0.40, depressive symptoms: *r* = −0.57) were comparable to those at Wave 10 (self-esteem: *r* = 0.45, loneliness: *r* = −0.41, depressive symptoms: *r* = −0.59), pointing toward construct continuity from adolescence through young adulthood.

##### Self-esteem

Self-esteem was assessed using three items (e.g., “I like myself just the way I am”) which were derived from Rosenberg’s (1965) original 10-item self-esteem scale. Item reduction was based on factor and reliability analyses (see Scales Manual; Thönnissen et al. 2019) and the final measure was found to be a good proxy for the full scale (M. D. Johnson et al. 2017). To further ensure that the measure assessed the same construct over time, measurement invariance was evaluated. Constraining the factor loadings and intercepts for each indicator to be equal across measurement occasions (i.e., strong measurement invariance) resulted in a good fit (Root mean square error of approximation [RMSEA] = 0.06, comparative fit index [CFI] = 0.99, Tucker-Lewis index [TLI] = 0.99) suggesting that the measurement of self-esteem was consistent over time. The response format ranged from 1 (*not at all*) to 5 (*absolutely*). Cronbach’s alpha was 0.69.

##### Loneliness

Loneliness was assessed using a single item (i.e., “I feel lonely”), which was derived from the UCLA loneliness scale (Russell et al. 1980). Response options ranged from 1 (*not at all*) to 5 (*absolutely*). A direct single-item measure is used frequently in loneliness research and has been shown to be both reliable (Zhong et al. 2016) and to converge very well with studies using multiple items (Mund and Neyer 2019). The correlations between loneliness and the other psychosocial adjustment variables at Wave four (life satisfaction: *r* = −0.40, self-esteem: *r* = −0.45, depressive symptoms: *r* = 0.51) were comparable to those at Wave 10 (life satisfaction: *r* = −0.41, self-esteem: *r* = −0.50, depressive symptoms: *r* = 0.58), suggesting construct continuity across measurements.

##### Depressive symptoms

The Depressive Symptoms Scale included 10 items from the State-Trait-Depression Scale (STDS Form Y-2; Spaderna et al. 2002), five of them assessing negative mood in general (e.g., “I am sad”) and the other five assessing positive mood (e.g., “I enjoy life”). To obtain the mean score of depressive symptoms, the items assessing positive mood were recoded. Imposing strong measurement invariance resulted in a good fit (RMSEA = 0.05, CFI = 0.97, TLI = 0.98), indicating no differences in measurement across time. The response options ranged from 1 (*almost never*) to 4 (*almost always*). Cronbach’s alpha was 0.86.

### Data Analyses

#### Latent profile analysis

Latent profile analysis (LPA) was conducted to identify groups of adolescents differing in their romantic experiences. LPA is a person-centered approach that identifies classes of individuals characterized by similar responses on a set of continuous indicator variables (Nylund-Gibson and Choi 2018). In the present study, the three variables of romantic involvement were used as indicators of class membership. Participants who indicated being single during the study or who reported having had their first relationship after the age of 20 were categorized a priori into the singles group and were, therefore, not considered in this analysis.

Models ranging from two to six classes were evaluated and the optimal model with regard to the number of classes was determined based on fit statistics such as the Akaike Information Criterion (AIC), consistent AIC (CAIC), Bayesian Information Criterion (BIC), and sample-size adjusted BIC (aBIC). For all four statistics, lower values indicate a better model fit. When there is no global minimum, the point with diminishing returns can be used to choose the optimal number of classes (Nylund-Gibson and Choi 2018). Results from the Lo-Mendell-Rubin likelihood ratio test (LMR), and the bootstrap likelihood ratio test (BLRT) were also considered, both of which indicate whether the solution with *k* classes fits the data better than a solution with *k* − 1 classes. In addition, entropy was examined, where higher values indicate higher classification accuracy. Lastly, sample size of the smallest group was considered; this was done to avoid overfitting and to establish sufficient power for further analyses.

Analyses were carried out with Mplus, version 7.4 (Muthén and Muthén 2010). 500 sets of starting values and a 50 final stage optimization for each model was used to avoid local solutions of maximum likelihood (Geiser 2010). Class-specific means were freely estimated, while class-specific variances were constrained to be equal. Once the best fitting model was selected, individuals were assigned to groups based on their highest affiliation probability.

#### First-order latent basis growth curve models

To test whether the previously defined groups differed in their psychosocial adjustment in middle adolescence and in their psychosocial development through young adulthood, a series of first-order latent basis growth curve models was calculated (Grimm et al. 2017).

First-order latent basis growth curve models were calculated separately for each of the four psychosocial adjustment variables. For each model, one manifest indicator variable from every available measurement occasion (either mean or single-item value) was used. For the slope, the first slope-loading was fixed to zero and the last slope-loading to one to identify the direction and degree of change from the first to the last measurement point. The slope loadings in between were freely estimated. The variances and covariance of the latent intercept and slope factor were also freely estimated. For the manifest variables, the means were fixed to zero and variances were set to equal across all measurement occasions and variances.

In order to test group differences, group affiliation was tested as a time-invariant predictor variable of the intercept and slope factor. Group affiliation was included as a dummy-coded variable with varying reference groups, so that each group was compared with one another. To account for multiple significance tests and to avoid Type 1 error rates, only effect sizes (Cohen’s *d*) larger than |0.10| with *p* values lower than 0.01 (Mund and Neyer 2014) were interpreted.

Model fit indices such as RMSEA, CFI, and TLI were evaluated. RMSEA values < 0.05 and CFI & TLI values >0.95 were considered to indicate a good model fit (Grimm et al. 2017). All models were calculated with a structural equation modeling approach using the *lavaan* package (Rosseel 2012) in R (R Core Team 2019).

Missing values among the psychosocial adjustment variables ranged from 3.34% in self-esteem at Wave three to 43.55% in loneliness at Wave 10. In order to use all available information to compute the model parameters, the full information maximum likelihood procedure (FIML) was used to handle missing data. This procedure provides efficient estimation of the parameters and is less biased than listwise deletion, pairwise deletion, or mean imputation in longitudinal research (Enders 2010). Furthermore, FIML has been shown to perform well in cases with large proportions of missing data, especially when the missing-at-random-assumption is met (D. R. Johnson and Young 2011), which was the case in the present study.

## Results

### Descriptive Results

Descriptive statistics and correlations between study variables are reported in Table 2. Participants who were romantically active in their adolescence reported having had their first relationship in middle adolescence, had more than one romantic relationship on average, and spent around 24 total months of their adolescence in romantic relationships. These three variables were significantly correlated with each other: The younger participants were at their first relationship, the more partners and the longer the total length of romantic involvement they reported by age 20.

**Table 2:** Descriptive characteristics and pearson correlations among the study variables

Variable	1	2	3	4	5	6	7
Romantic involvement							
1. Age of first relationship	–	–	–	–	–	–	–
2. Number of partners	*−*0.54***	–	–	–	–	–	–
3. Months in relationships	*−*0.53***	0.36***	–	–	–	–	–
Psychosocial adjustment							
4. Life satisfaction	0.03	*−*0.04	0.03	–	–	–	–
5. Self-esteem	0.03	*−*0.03	*−*0.04	0.61***	–	–	–
6. Loneliness	*−*0.06*	0.00	*−*0.05*	*−*0.50***	*−*0.61***	–	–
7. Depressive symptoms	*−*0.02	0.03	0.02	*−*0.68***	*−*0.76***	0.65***	–
*M*	16.25	1.93	24.31	7.75	3.91	2.09	1.75
*SD*	1.85	1.12	16.48	1.02	0.65	0.85	0.37

The correlations regarding the psychosocial adjustment variables refer to the average values across all waves

*p < 0.05; ***p < 0.001

The psychosocial adjustment variables were also all significantly correlated with each other: Both the correlations between life satisfaction and self-esteem and between loneliness and depressive symptoms were positive. In evaluating the correlations between romantic relationship indicators and psychosocial adjustment, loneliness was found to be related to two of the indicators: The later participants started dating and the more time they spent in relationships, the less lonely they felt.

### Latent Profile Analysis

Table 3 presents the fit statistics for the LPA models ranging from two to six classes. The model with six classes could not be properly identified, as the best log likelihood values in the model estimation could not be replicated and estimates were unreliable. Out of the remaining models, the three-class solution was chosen for the final model for the following four reasons: First, although each fit statistic decreased across the two- to the five-class solution, the smallest decrease was found when moving from the three- to the four-class solution, suggesting minimal improvement when a fourth class was included. Second, the LMR comparing the three- to the four-class model was not significant, again suggesting that a model with four classes did not fit the data better than the model with three classes. Third, beyond the solution of three classes, the sample size of the smallest group lay below the 5% minimum recommended by Nylund-Gibson and Choi (2018), as this would limit statistical power for further analyses. Fourth, when comparing the distribution of romantic relationship indicators in the three- and the four-class solutions, the additional fourth class was found to be conceptually redundant to one of the other three classes.

**Table 3:** Fit statistics for LPA models ranging from two to six classes

Model	AIC	CAIC	BIC	aBIC	LMR	BLRT	Entropy	Min. class
2 classes	28632	28697	28687	28656	*p* < 0.001	*p* < 0.001	0.69	42.19%
**3 classes**	**28140**	**28232**	**28218**	**28174**	***p*** = **0.015**	***p*** < **0.001**	**0.77**	**9.14%**
4 classes	27854	27972	27954	27897	*p* = 0.404	*p* < 0.001	0.83	3.08%
5 classes	27380	27525	27503	27433	*p* = 0.022	*p* < 0.001	0.95	3.08%
6 classes	Model was not well identified^a^							

Bold font indicates the selected model

*LPA* Latent profile analysis, *AIC* Akaike information criterion, *CAIC* Consistent AIC, *BIC* Bayesian information criterion, *aBIC* Sample-size adjusted BIC, *LMR* Lo-Mendell-Rubin likelihood ratio test, *BLRT* Bootstrap likelihood ratio test, *Min. class* % of smallest class based on the individuals’ highest affiliation probability

^a^The model estimation could not be replicated and consequently, the estimates were untrustworthy

After deciding on the final model, individuals were assigned to classes based on the highest affiliation probability. The entropy score for the final model indicated good classification accuracy. In addition to the three classes covering romantic involvement during adolescence, a fourth class was included for those participants who remained single during their adolescence. Descriptive information on the four classes is shown in Table 4. The final number of classes was in line with the first hypothesis.

**Table 4:** Descriptive information of the classes

Variable	Late starters	Moderate daters	Frequent changers	Singles
Sample size	907	833	175	542
% females	0.48	0.58	0.50	0.40
Romantic involvement				
Age of first rel. *M* (*SD*)	17.67 (1.29)_a_	15.12 (1.18)_b_	14.32 (1.37)_c_	–
No. of partners *M* (*SD*)	1.19 (0.40)_a_	2.20 (0.69)_b_	4.5 (0.88)_c_	0 (0)
Months in rel. *M* (*SD*)	13.83 (10.25)_a_	33.56 (15.43)_b_	34.99 (14.37)_b_	0 (0)

*rel.* relationships

Means with different subscripts within a row are significantly different from one another (*p* < 0.05) as tested with a Tukey’s post-hoc test

A MANOVA comparing the three classes from the LPA with regard to romantic experiences proved to be significant, Wilks’ Λ = 0.16, *F*(2, 1903) = 969.81, *p* < 0.001. Tukey’s post hoc test revealed significant differences between the three groups on all of the romantic relationship variables: age of first relationship, *F*(2, 1912) = 1138, *p* < 0.001, number of romantic partners, *F*(2, 1912) = 2425, *p* < 0.001, and romantic involvement in months, *F*(2, 1903) = 552.5, *p* < 0.001.

The first and largest class was labeled *late starters* (36.91%) because this group was characterized by a later age of entering the first romantic relationship than all the other groups (all *p* < 0.001). In addition, this group showed the lowest number of partners and lowest overall romantic involvement duration (all *p* < 0.001).

The smallest group was called *frequent changers* (7.12%). This group presented the highest number of romantic partners among all groups (all *p* < 0.001) and was also characterized by the earliest age of entering one’s first relationship (all *p* < 0.001).

The second-largest group comprised 33.90% of the sample and was named *moderate daters*, as this group lay in between the late starters and frequent changers. Although this group spent as many months in romantic relationships as the frequent changers (*p* = 0.390), participants in this group started dating at a later age (*p* < 0.001) and had significantly fewer partners (*p* < 0.001) than the frequent changers. However, they started at an earlier age (*p* < 0.001) and reported more partners (*p* < 0.001) than the late starters.

The group of those who reported not having had a romantic relationship by age of 20 was termed *continuous singles* and comprised 22.06% of participants.

Although these four groups differed with regard to sex, χ^2^(3) = 33.19, *p* < 0.001—More female participants were classified into the moderate daters group and more male participants into the singles group—the effect size was small (Cramer’s *V* = 0.13).

### First-Order Latent Basis Growth Curve Models

Results of the latent basis growth curve models for each outcome separated by group can be found in Table 5.

**Table 5:** Parameter estimates of the latent growth curve models for each outcome separated by group

Variable	Late starters			Moderate daters			Frequent changers			Singles		
	*b*	*SE*	*p*	*b*	*SE*	*p*	*b*	*SE*	*p*	*b*	*SE*	*p*
Life satisfaction												
Intercept W1	7.95	0.04	<0.001	8.06_a_	0.04	<0.001	7.79	0.09	<0.001	7.77_b_	0.05	<0.001
Slope	*−*0.27	0.05	<0.001	*−*0.36	0.05	<0.001	*−*0.39	0.11	<0.001	*−*0.38	0.06	<0.001
Intercept W10	7.68_a_	0.04	<0.001	7.70_a_	0.05	<0.001	7.40	0.09	<0.001	7.40_b_	0.05	<0.001
Self-esteem												
Intercept W2	3.99	0.03	<0.001	3.99	0.03	<0.001	3.93	0.06	<0.001	3.93	0.03	<0.001
Slope	*−*0.12	0.03	<0.001	*−*0.11	0.03	<0.001	*−*0.20	0.07	0.003	*−*0.13	0.04	0.001
Intercept W10	3.87	0.03	<0.001	3.88	0.03	<0.001	3.73	0.06	<0.001	3.80	0.03	<0.001
Loneliness												
Intercept W4	2.05_a_	0.03	<0.001	1.93_a_	0.04	<0.001	2.06	0.08	<0.001	2.24_b_	0.04	<0.001
Slope	0.01	0.04	0.792	0.16	0.05	<0.001	0.14	0.10	0.160	0.15	0.06	0.008
Intercept W10	2.06_a_	0.04	<0.001	2.09_a_	0.04	<0.001	2.20	0.08	<0.001	2.39_b_	0.05	<0.001
Depressive symptoms												
Intercept W2	1.68	0.01	<0.001	1.65	0.01	<0.001	1.72	0.03	<0.001	1.68	0.02	<0.001
Slope	0.14	0.02	<0.001	0.16	0.02	<0.001	0.17	0.03	<0.001	0.16	0.02	<0.001
Intercept W10	1.82	0.02	<0.001	1.80	0.02	<0.001	1.89	0.03	<0.001	1.84	0.02	<0.001

The first intercept represents the initial level of the variable at the first wave when variable was assessed. The second intercept refers to the level at the last measurement point. Slope represents change across measurement occasions. Means with different subscripts within a row are significantly different from one another (*p* < 0.01)

#### Life satisfaction

The latent growth curve model for life satisfaction provided a good fit with RMSEA = 0.04, CFI = 0.95, and TLI = 0.95. With regard to initial group differences in life satisfaction, continuous singles tended to show lower levels of initial life satisfaction compared to those in the moderate daters group (*d* = −0.29, *p* < 0.001). There were no other significant differences in the intercepts between the groups. The slope across measurements was significantly negative for each group, suggesting a general decrease in life satisfaction over time. In young adulthood, the significant difference between singles and moderate daters was still present (*d* = −0.29, *p* < 0.001) and in addition, singles also reported lower life satisfaction than the late starters in young adulthood (*d* = −0.27, *p* < 0.001).

#### Self-esteem

The latent growth curve model for self-esteem suggested a good fit (RMSEA = 0.04, CFI = 0.98, TLI = 0.97). Results indicated no significant group differences in either the intercepts or the slopes. Independent of group affiliation, self-esteem decreased significantly across the years.

#### Loneliness

The model fit for loneliness was good with RMSEA = 0.03, CFI = 0.98, and TLI = 0.98. Group comparisons indicated higher initial loneliness levels among the continuous singles compared to both the moderate daters (*d* = 0.43, *p* < 0.001) and late starters (*d* = 0.26, *p* < 0.001). These differences were also present in young adulthood with *d* = 0.38, *p* < 0.001 for continuous singles vs. moderate daters, and *d* = 0.41, *p* < 0.001 for continuous singles vs. late starters, respectively. The magnitude of change in loneliness was equal in all groups and indicated a general increase in loneliness over time.

#### Depressive symptoms

The RMSEA = 0.05, CFI = 0.96, and TLI = 0.95 suggested a good fit for the latent growth curve model for depressive symptoms. Adolescents’ depressive symptoms and their change over time did not differ across groups. In all groups, depressive symptoms increased over time through young adulthood.

Together, these results suggest that part of the hypothesis regarding the link between romantic involvement and psychosocial adjustment was supported. In particular, those with no romantic experiences in adolescence indicated lower life satisfaction and more loneliness than those who dated moderately. However, the group characterized by frequent dating did not differ from the others in these aspects and no group differences were found in self-esteem or depressive symptoms. Regarding the exploratory question, although romantic involvement had no impact on the rate of change of psychosocial adjustment, lasting associations were found for life satisfaction and loneliness in young adulthood.

### Sensitivity Analyses

Two sensitivity analyses were conducted to test the robustness of the results. First, parametric bootstrapping with 1000 replications was applied to the growth curve models. The results of these analyses fully replicated those of the main analyses. Second, to ensure that findings were not sensitive to missing data, all models were further estimated utilizing solely participants with complete data. Again, the results of the main analyses were largely confirmed. All statistically significant effects found in the main analyses were fully replicated. However, two additional group differences emerged: Late starters reported significantly higher life satisfaction than continuous singles (*d* = 0.36, *p* < 0.001) at the first wave, and continuous singles reported more depressive symptoms than the moderate daters (*d* = 0.30, *p* = 0.001) at the last wave.

Overall, the results of these sensitivity analyses largely replicated the main findings. In the following section, only the robust results of the main analyses will be discussed. Finally, it should be noted that all data analyses were conducted as planned and no participants or variables were excluded due to lack of significance.

## Discussion

Romantic relationships represent a new and developmentally important context for adolescents (Furman and Shaffer 2003). However, not all adolescents have the same romantic experiences and there is large variation in the age at which adolescents first start dating and how romantically active they are (Collins et al. 2009). Further, those characterized by either being overly romantically involved or by having little to no relationship experience may be especially prone to experiencing poorer adjustment in both adolescence and young adulthood. Using data from a German representative longitudinal study, the current study identified four groups of adolescents based on their romantic involvement between the ages of 10 and 20 and tested whether they differed in their psychosocial adjustment from middle adolescence through young adulthood. These four groups included late starters, moderate daters, frequent changers, and continuous singles. The continuous singles reported lower life satisfaction and higher loneliness compared to the moderate daters and late starters. This effect was not only evident in middle adolescence but remained over a period of 10 years through young adulthood.

### Variability in Romantic Experiences During Adolescence

Although both scholars and lay culture often assume adolescent romantic relationships to be short and trivial, these findings suggest great variability in romantic relationship experiences with regard to the age when adolescents first get involved, how many partners they have, and how much total time they spend in these relationships. Late starters and moderate daters were similar in their group sizes and represented the largest groups, whereas only a few adolescents were categorized as frequent changers. Most adolescents started dating in middle and late adolescence, had around one to two different partners, and were romantically involved for a total of around 14 to 34 months.

By using multiple indicators of romantic involvement as well as covering the whole period of adolescence from early to late adolescence in a large and representative sample, the current study replicates and augments the findings of previous studies (Boisvert and Poulin 2016; Connolly et al. 2013; Orpinas et al. 2013), which identified similar groups and group proportions. The period of adolescence seems to be marked by great variability in relationship experiences, and including those who did not date at all during their adolescence showed that a substantial proportion of adolescents are not romantically active in their youth. With 22% of a representative sample of adolescents, singles account for a nontrivial proportion of adolescents that needs to be considered to obtain a comprehensive understanding of romantic activities (or lack thereof) during this important period of life.

### Concurrent Effects on Psychosocial Adjustment

Previous findings regarding romantic involvement during adolescence and its effect on psychosocial adjustment have been mixed, stressing both risks and opportunities. Out of the four investigated aspects of adjustment, group differences were found in two: Moderate daters reported higher life satisfaction than the continuous singles in middle adolescence, and both moderate daters and late starters felt less lonely than the continuous singles in late adolescence.

That the moderate daters and late starters indicated better adjustment than the continuous singles (at least in some aspects) was in line with the hypothesis, as both groups could be assumed to represent groups of adolescents with normative relationship experiences with regard to age of first romantic experience and overall romantic involvement (as compared to the abstaining group). The differences found in life satisfaction and loneliness could reflect the social nature of romantic involvement. For many adolescents, dating is a way to achieve social status and validation from peers (Carlson and Rose 2007), and having a romantic partner has been identified as a consistent factor shielding against loneliness (Luhmann and Hawkley 2016). Those who remain single during their adolescence might feel as though they are missing out on these pleasant and enriching social experiences, which could make them less satisfied with their lives and more prone to feeling lonely.

Self-esteem and depressive symptoms, on the other hand, were entirely independent of relationship experiences during adolescence. Both loneliness and life satisfaction may therefore represent more context-dependent aspects of psychosocial adjustment that are more easily affected by changes in relationship status. It is important to note at this point, however, that psychosocial adjustment was assessed first in middle to late adolescence. It could be that continuous singles were already less satisfied and more lonely in childhood and early adolescence, which could have prevented them from engaging in a romantic relationship in the first place.

The lack of differences between the other groups of romantically active adolescents was surprising. Based on the theoretical frameworks outlined in the introduction, as well as previous findings showing that early age of first initiation (Connolly et al. 2013; Natsuaki and Biehl 2009) and accumulation of romantic partners (Davies and Windle 2000; Davila 2008) were associated with more adjustment problems, the group of frequent changers was expected to show lower levels of adjustment compared to moderate daters and late starters. The frequent changers were also likely to having experienced the most break-ups compared to the other groups, an event that has been found to be a prospective risk factor for psychological distress (Rhoades et al. 2011). The authors offer two possible explanations for the lack of group differences concerning the frequent changers: First, compared to findings of previous studies, frequent changers initiated dating at a later age (i.e., middle adolescence), when the consequences of being in a relationship and experiencing breakups may be less pronounced than in early adolescence. Second, although frequent changers experienced more relationship dissolution than their peers, their relationships were also likely to be of short duration and of lower commitment, which may have alleviated the impact of each breakup on mental well-being. These explanations are, however, speculative, and should be explored in further research.

### Prospective Effects on Psychosocial Adjustment

The group differences found in adolescence were also present in young adulthood, with continuous singles reporting lower life satisfaction and higher loneliness than the moderate daters. In young adulthood, additional group differences emerged: While late starters did not differ in life satisfaction from continuous singles during adolescence, they reported higher life satisfaction in young adulthood. This finding could reflect the beneficial effect of engaging in one’s first relationship for later well-being, and would be in line with findings from Wagner et al. (2015), who found an increase in life satisfaction among young adults who entered into their first relationship.

There were no group differences regarding the rate of change in any of the adjustment variables. In general, however, a negative trend was observed: Life satisfaction and self-esteem decreased from middle adolescence through young adulthood, and loneliness and depressive symptoms increased, regardless of adolescents’ romantic relationship experiences. Together with previous findings showing a normative trend of decreasing life satisfaction (Goldbeck et al. 2007) and increasing depressive symptoms (Thapar et al. 2012) during adolescence, the current findings suggest that adolescence is a critical period of psychosocial development. This could be, at least in part, explained by the many developmental challenges that adolescents face during this time, including biological, cognitive, social, and academic transitions (Steinberg 2005), and calls for further investigation concerning potential risk and protective factors that go beyond the field of romantic involvement.

Together, the analyses of the current study provide compelling evidence that getting romantically involved is a normative event during adolescence and that there is great variability in the romantic experiences adolescents have. Further, a substantial proportion of adolescents do not date, and the present findings highlight the importance of taking into account this often ignored group when examining romantic relationship patterns. Continuous singles are particularly interesting, as they seem to differ from all other groups with regard to some aspects of psychosocial adjustment both in adolescence and through young adulthood. It should be noted, however, that although singles indicated lower life satisfaction and more loneliness than the other groups with small to moderate differences (|0.26| *≤* *d*s ≤ |0.43|), mean values lay within a clinically unremarkable range. Nevertheless, these differences may have significant long-term consequences for well-being.

### Limitations and Future Directions

These findings should be interpreted in light of some limitations. First, although the study covered romantic relationship experiences starting at age 10, the first assessments of psychosocial adjustment took place in middle to late adolescence (age 16–19). This asynchronous design implies limits regarding causal interpretation of the results. However, the finding that late starters only differed in their life satisfaction from the continuous singles in young adulthood, not in adolescence, provides a first hint that engaging in one’s first romantic relationship might bring increased life satisfaction (see also Wagner et al. 2015). Future research should assess both romantic involvement and adjustment beginning in early adolescence to disentangle possible selection and socialization effects of romantic relationships.

Second, loneliness and life satisfaction were assessed using single-item measures and self-esteem with an abbreviated measure consisting of three items. Although these measures were shown to perform well psychometrically, and although the use of short measures is common in large-scale panel studies to reduce participants’ burden, abbreviated measures are probably less precise compared to full-scale multiple-item measures. Future studies should therefore replicate the current findings with multi-item measures.

Third, some of the information on romantic involvement was assessed retrospectively, as adolescents reported on both current and past relationship experiences. Although the EHC is a reliable tool to assess retrospective events that happened within the past year (Glasner and Van Der Vaart 2009), reports on more remote events (i.e., between age 10 until the first assessment point) might have been less accurate. Future studies should therefore ensure shorter intervals for retrospective reporting of romantic experiences.

Finally, psychosocial adjustment may be affected by factors related to romantic involvement such as relationship quality (Madsen and Collins 2011) and sexual experiences (Williams et al. 2008), which were not included in the present study. Future research may benefit from exploring how relationship quality and sexual behavior are associated with level and change of adjustment from adolescence through young adulthood while controlling for age of initiation, number of partners, and total time spent in relationships.

## Conclusion

Although most people engage in dating during their adolescent years, there is high variation in when they do so and how romantically active they are. Given the centrality of romantic relationships in adolescence, understanding the significance of those different experiences for psychosocial adjustment is important. The present study covered multiple aspects of romantic experiences from early to late adolescence in a large and representative longitudinal study and connected them to both positive and negative aspects of psychosocial adjustment over a period of 10 years. The findings revealed that variability in romantic relationship experiences is the norm rather than the exception during this period: Although most adolescents started dating in middle to late adolescence and were engaged in more than one relationship, a substantial proportion did not date during their adolescence. Those continuous singles were less satisfied with their lives and felt lonelier compared to the moderate daters both during adolescence and young adulthood. Together, these findings provide a comprehensive picture about the diversity of relationship experiences during adolescence and highlight their role in the short- and long-term psychological well-being of youth.
